# Insights into the ameliorative effect of ZnONPs on arsenic toxicity in soybean mediated by hormonal regulation, transporter modulation, and stress responsive genes

**DOI:** 10.3389/fpls.2024.1427367

**Published:** 2024-07-30

**Authors:** Muhammad Zeeshan, Chenyu Sun, Xin Wang, Yuxin Hu, Hao Wu, Shengnan Li, Abdul Salam, Shiqi Zhu, Aamir Hamid Khan, Paul Holford, Mohammad Ajmal Ali, Mohamed Soliman Elshikh, Zhixiang Zhang, Peiwen Zhang

**Affiliations:** ^1^ National Key Laboratory of Green Pesticide, South China Agricultural University, Guangzhou, China; ^2^ Yingdong College of Biology and Agriculture, Shaoguan University, Shaoguan, China; ^3^ College of Natural Resources and Environment, Northwest A&F University, Yangling, China; ^4^ College of Pastoral Agriculture Science and Technology, Lanzhou University, Lanzhou, China; ^5^ Faculty of Biology and Environmental Protection, Department of Biogeography, Paleoecology and Nature conservation, University of Lodz, Lodz, Poland; ^6^ School of Science, Western Sydney University, Penrith, NSW, Australia; ^7^ Department of Botany and Microbiology, College of Science, King Saud University, Riyadh, Saudi Arabia

**Keywords:** abiotic stress, heavy metal, hub genes, *Glycine max*, WGCNA

## Abstract

Arsenic (As) contamination of agricultural soils poses a serious threat to crop productivity and food safety. Zinc oxide nanoparticles (ZnONPs) have emerged as a potential amendment for mitigating the adverse effects of As stress in plants. Soybean crop is mostly grown on marginalized land and is known for high accumulation of As in roots than others tissue. Therefore, this study aimed to elucidate the underlying mechanisms of ZnONPs in ameliorating arsenic toxicity in soybean. Our results demonstrated that ZnOB significantly improved the growth performance of soybean plants exposed to arsenic. This improvement was accompanied by a decrease (55%) in As accumulation and an increase in photosynthetic efficiency. ZnOB also modulated hormonal balance, with a significant increase in auxin (149%), abscisic acid (118%), gibberellin (160%) and jasmonic acid content (92%) under As(V) stress assuring that ZnONPs may enhance root growth and development by regulating hormonal signaling. We then conducted a transcriptomic analysis to understand further the molecular mechanisms underlying the NPs-induced As(V) tolerance. This analysis identified genes differentially expressed in response to ZnONPs supplementation, including those involved in auxin, abscisic acid, gibberellin, and jasmonic acid biosynthesis and signaling pathways. Weighted gene co-expression network analysis identified 37 potential hub genes encoding stress responders, transporters, and signal transducers across six modules potentially facilitated the efflux of arsenic from cells, reducing its toxicity. Our study provides valuable insights into the molecular mechanisms associated with metalloid tolerance in soybean and offers new avenues for improving As tolerance in contaminated soils.

## 1 Introduction

Soybean [*Glycine max* (L.) Merrill] belongs to the Fabaceae and accounted for ~60% of world oilseed production in 2020/21 (SoyStats, 2023). The crop had the fourth largest area of cultivation of all crops worldwide in 2021 and ranked seventh largest in terms of production (FAOSAT, www.fao.org). Soybean is thought to have originated in China (Lee et al., 2011) and is commonly known as the “miracle crop”, as it is a rich source of high-quality oils and proteins containing 20 and 40% of these compounds, respectively (Clemente and Cahoon, 2009). The southern provinces of China belong to the tropical/subtropical, multi-season ecological zone and are considered suitable for soybean cultivation. However, mining activities in the area have led to pollution of the soil with arsenic (As) and other metals and/or metalloids, causing reduced production and endangering food safety.

Soybean crops are largely cultivated on marginalized and contaminated land (Vezza et al., 2022), which not only limits productivity but also facilitates the accumulation of metal(loids) into the food chain (Majumdar et al., 2021). Studies have reported that As-contaminated underground water is often used to irrigate soybean crops in many regions (Mariño et al., 2020; Singh and Srivastava, 2020). Arsenate (As(V)) is the predominant species of inorganic As in soils and enters root cells through the activity of phosphate (Pi) transporters due to their chemical structure similarity (Zhao et al., 2010). Its toxicity arises because it replaces phosphate ions in ATP synthesis, depriving cells of energy (Armendariz et al., 2016). Upon uptake, most As is accumulated in roots, then shoots (Wu et al., 2020). As contents in different parts of rice plants follows the order roots > stems and leaves > husks > grain (Seyfferth et al., 2016). The mechanisms of As uptake from the soil and long-distance transport to aerial plant tissue and detoxification are well known (Zhao et al., 2010; Zvobgo et al., 2018b). For example, multidrug and toxic compound extrusion (MATE), natural resistance-associated macrophage protein (NRAMP), and ATP-binding cassette (ABC) family transporters are reported to have a role in As uptake and distribution in different higher plant species (Yanitch et al., 2017; Zvobgo et al., 2018b).

Nano-enabled agrochemicals have attracted increasing interest due to their potential applications in the management of plant stress as sustainable alternatives to other techniques (Agathokleous et al., 2020). For example, seed priming with zinc oxide nanoparticles (ZnONPs) potentially improved the growth and biomass production of maize (Salam et al., 2022). Similarly, supplementation with ZnONPs improved the metal tolerance index, reduced As uptake, and promoted the accumulation of antioxidants in *Oryza sativa* (Wang et al., 2018; Wu et al., 2020). Studies have shown that ZnONPs release Zn^2+^ ions (Horie et al., 2012; Hu et al., 2013), and an antagonistic interaction between As(V) and Zn was observed when both were applied to a wheat crop (Gong et al., 2020). As(V) and Zn have been shown to be taken up by inorganic phosphate transporters (*PHT1*) (Huang et al., 2000; Jain et al., 2013; Khan et al., 2014). In this context, there is a need for investigating the mutual interaction of ZnONPs and AsV in soybean.

A number of studies have been made on the physiological mechanisms of tolerance to heavy metal(loids), including cobalt (Co) in *Zea mays* (Salam et al., 2022) and As in *Oryza sativa* (Wu et al., 2020) and soybean (Zeeshan et al., 2021, 2022) induced by ZnONPs. These studies have shown that supplementation with ZnONPs ameliorates reductions in chlorophyll and photosynthesis, maintains the integrity of membranes by reducing oxidative damage, and increases nutrient uptake. ZnONPs have also been shown to regulate phytohormones in *Arabidopsis* (Vankova et al., 2017) and As(V) to modulate levels in soybean (Vezza et al., 2022). This suggests the involvement of phytohormone signaling in the mitigation of metalloid stress by nanoparticles (NP). In addition, several metal homeostasis genes, such as zinc finger family proteins, heavy metal ATPase4 (HMA4), and heavy metal transport/detoxification superfamily proteins, have been found to be regulated upon individual applications of ether As(V) or Zn in barley (Huang et al., 2012; Zvobgo et al., 2019) and *Arabidopsis* (Landa et al., 2015). However, to our knowledge, no studies at the molecular level have been performed examining the mitigating effects of ZnONPs on As intoxication. In addition, compared to rice and *Arabidopsis*, transcriptomic data of soybean roots are scarce, and a systematic examination of the effects elicited by the ZnONPs on plant physiology at the molecular level under As stress is lacking.

The recent release of a transcriptomic atlas and the complete sequencing of the genome of the variety “Williams 82” (Libault et al., 2010; Schmutz et al., 2010) have opened new windows for research on soybean functional genomics. Therefore, to uncover the protective effects of ZnONPs on As(V) stress, the present study first focused on investigating the role of ZnONPs as a nano-fertilizer on root architecture, plant biomass, photosynthetic attributes, hormonal regulation and As(V) uptake under As(V) stress in soybeans. We then examined the abundance of individual mRNAs of the whole transcriptome to identify differentially regulated genes. These data were subjected to weighted gene co-expression network analysis (WGCNA) to identify genes whose activities were coordinated into multi-gene, adaptive complexes (modules/clusters) thus gaining new insights into the effects of As(V) on transcription and the mechanisms by which ZnONPs mitigate its toxic effects.

## 2 Materials and methods

### 2.1 Experimental design and treatment detail

Soybean seeds (genotype ZhongHoung302) were germinated in sterile vermiculite for ten days. Uniform seedlings were then transferred to 10 L pots filled with modified half-strength Hoagland’s solution as described by Sugiyama et al. (2016) and grown under ambient temperatures of 25–28°C and a 15 h/9 h day/night cycle with artificial lighting. When the plant reached the V2 growth stage (first two trifoliolate leaf nodes), the nutrient solutions were either left unsupplemented or supplemented with either 25 μmol L^−1^ of arsenate (Na_2_HAsO_4_) only or with arsenate plus ZnONPs at 25 μmol L^−1^ or 50 μmol L^−1^, hereafter referred to as CK, AsV-only, ZnOA, and ZnOB, respectively. The unsupplemented pots receiving only nutrient solution were considered as controls. The Na_2_HAsO_4_ and ZnONPs were purchased from Sigma Aldrich, USA and used as received. The size of ZnONPs was 20 nm and their zeta potential was -16 to 23 mV in an aqueous solution as determined by a zeta potential analyzer (NanoBrook, Brookhaven, USA). The ZnONPs were characterized by X-ray diffraction and energy dispersion spectra mapping as described in Zeeshan et al. (2021). The stock solution of ZnONPs, (4.07 mg L^−1^) was suspended in ddH_2_O and the suspension was stirred using ultra-sonicator for 1 h to disperse the NPs before use. In addition, the pots in which the seedlings were grown were regularly stirred to discourage NP aggregation and to maximize their suspension in the nutrient solution. The pH of the nutrient solution was kept at ~5.8, and the solution was changed twice a week. After 10 days of treatment, data were recorded with respect to various physiological parameters, and the roots harvested, thoroughly washed with ddH_2_O and assessed as described below then frozen in liquid nitrogen and stored at −80°C until determination of hormones and the extraction of RNA.

### 2.2 Observation of root phenotypes and determination of dry matter accumulation, relative water contents, and total As contents

After 10 days of treatment, roots samples were collected, scanned with an Epson Perfection V500 photo scanner (Nagano, Japan), and the root total lengths, root diameters and the number of lateral and secondary roots were measured using WinRhizo Pro (S) v. 2009a software (Regent Instruments Inc., Quebec City, QC, Canada). Dry matter (DM) accumulation by the seedlings was measured after drying the whole plant at 70°C for three days. Relative water contents (RWC) in fully expanded trifoliolate leaves were determined following procedure mentioned by Zeeshan et al. (2020).

Whole soybean seedlings were collected and separated into roots and shoots. The roots were placed in 0.01 M ethylenediamine tetra acetic acid for 15 min and then carefully washed with ddH_2_O to remove As from the root surfaces. Then, the total As contents in the root samples was measured by atomic fluorescence spectrometry-mass spectrometry (AFS-MS). The samples were prepared by digesting 0.2 g dried roots samples in 5 mL of concentrated HNO_3_ (4 mL) and HCLO_4_ (1 mL) for 12 h at room temperature. A certified reference material [GBW10023 (GSB-14)] was used for calibration.

### 2.3 Photosynthetic efficiency, photosynthetic pigment contents and chlorophyll fluorescence

After treatment, changes in physiological factors affecting photosynthesis were determined by measuring gas exchange parameters and the contents of photosynthetic pigments. Parameters associated with photosynthesis (net photosynthesis (*Pn*), stomatal conductance (*Gs*), intercellular CO_2_ concentration (*Ci*), transpiration rate (*E*), chlorophyll fluorescence (*Fv/Fm*), and non-photochemical chlorophyll fluorescence quenching (NPQ) were recorded using a portable infrared analyzer (LI-6800 System; Li-COR) as described in Zeeshan et al. (2021). For the determination of pigment contents, fresh leaf samples were placed in 80% acetone at room temperature overnight in the dark. When the leaves became colorless, the extracts were centrifuged at 4000 × g for 12 min, and the absorbances of the supernatants were measured spectrophotometrically at 470, 663, and 646 nm from which chlorophyll *a* (chl *a*), chlorophyll *b* (chl *b*), and carotenoids concentrations were calculated following Lichtenthaler and Wellburn (1983).

### 2.4 Determination of endogenous phytohormones in root samples

The phytohormones, indole acetic acid (IAA), abscisic acid (ABA), gibberellic acid (GA_3_), and methyl jasmonate (MeJA), were assayed following the methods modified by Ahmad et al. (2022). Briefly, roots samples (50 mg) were ground to a powder using liquid nitrogen in a tissue homogenizer. The internal standards of the phytohormones, d_5_-IAA, d_6_-ABA, d_2_-GA_3_, and MeH_2_ JA, were made up in the extraction buffer that consisted of a ratio of 0.002:1:2 (v/v/v) of HCL, H_2_O, and 2-propanol and were added into the ground samples. The tubes containing samples were placed in a shaker for 30 min at 100 rpm and 4°C after which each tube was again shaken for 30 min at 4°C after the addition of 1 mL CH_2_Cl_2_. After centrifugation at 13,000 × g for 5 min, the supernatants were collected, dried with a nitrogen evaporator, and dissolved in 100 µL CH_3_OH for analysis by HPLC-MS. The equipment setup and related procedures are as reported in Ahmad et al. (2022).

### 2.5 RNA extraction, library preparation and sequencing

RNA was extracted from root tissue using TRIzol^®^ and purified from DNA contamination using RNase-free DNase I. The isolation of RNA was performed on two independent biological replicates for each treatment in the roots. RNA quality was verified using agarose gel electrophoresis and a Bioanalyser with quality control parameters. RNA concentration was assessed using a spectrophotometer. Library preparation and sequencing were then performed on a Majorbio sequencing platform following manufacturer’s instructions using Illumina technology. Library preparation steps included mRNA isolation using oligo(dT) beads, fragmentation in fragmentation buffer and synthesis of double-strand cDNA using random hexamer primers and a SuperScript double-stranded cDNA synthesis kit (Invitrogen, CA). The cDNAs were then processed with end repair mix, ‘A’ base addition, and phosphorylation. The sequences were randomly fragmented into small pieces of ~300 bp and amplified using Phusion DNA polymerase. Paired-end sequencing was performed on the Illumina NovaSeq 6000 sequencer following the manufacturer’s protocol.

### 2.6 Quality control of raw sequence data and reliability analysis

The raw sequencing data were filtered to obtain high-quality data (clean data) to ensure smooth subsequent bioinformatic analyses. This included the removal of connector sequences in reads and low-quality bases from both 3′ to 5′ and 5′ to 3′ (Q < 20) reads using Fastp (https://github.com/OpenGene/fastp). The filtered reads data were aligned by HISAT2 software (http://ccb.jhu.edu/software/hisat2/index.shtml, default parameters) to the *Glycine max* reference genome (version Wm82.a4; available at https://data.jgi.doe.gov/refine-download/phytozome?genome_id=508; accessed on Jan 10, 2023). The obtained aligned data/reads ranged from 86.58% to 93.32% of each library and were statistically analyzed by StringTie.

The reliability of the RNA-seq data was assessed using qRT-PCR, following the SYBR Green Mastermix protocol (Applied Biosystems, Waltham, MA, USA). A list of selected genes and gene specific primers used in this assay are provided in **Supplementary Table S1**. Primers were synthesized using primer premier 5.0 (Primer, Palo Alto, CA, USA). Two independent biological replicates and two technical replicates were used. The RNA templates were same to those previously used in library construction, whereas cDNA synthesis was carried out using the SuperMix First-Strand Synthesis kit (TransGen Biotech Co., Ltd, Beijing, China). The qRT-PCR assay was then performed and analyzed according to previously described methods (Zeeshan et al., 2021).

### 2.7 Differential expression analysis and functional enrichment

The expression levels of genes were quantified using RSEM software (http://deweylab.github.io/RSEM/) set with default parameters and were expressed as transcripts per million reads (TPM) to determine differentially expressed genes (DEGs) between samples. DEGs were identified using DESeq2 (http://bioconductor.org/packages/stats/bioc/DESeq2/) using the following screening criteria: *q*-values < 0.001 and |log2FC| ≧ 1; if a gene met both criteria, it was considered to be a DEG. To assign functional annotation to the identified genes, bioinformatic analyses such as Gene Ontology (GO) and Kyoto Encyclopedia of Genes and Genomes (KEGG) were performed using Diamond (GO, https://github.com/bbuchfink/diamond) and ID mapping (KEGG), respectively. Four other databases, NR, Pfam, COG/KOG, and SwissPort, were also searched for assigning functional annotations.

### 2.8 Construction of co-expression network and identification of hub genes

The identified DEGs were used for network construction and module identification using the R package “WGCNA”. We used TPM values of DEGs to determine the correlation strength between the nodes by calculating an adjacency matrix. A soft threshold of β = 10 was chosen to make the whole network fit the scale-free topology. Module partition and gene clustering were determined using a dynamic cutting algorithm. Principal components and Pearson correlation were applied to calculate the module eigengene and module-phenotypes associations, respectively. We selected the top 10 genes as hub genes in selected modules after calculating each gene connectivity within a module. The hub genes networks were visualized by Cytoscape (version 3.6.1). Finally, we conducted a GO pathway enrichment analysis to identify the biological functions of genes in selected modules.

### 2.9 Statistical analysis

The data for the physiological indexes and phytohormones were expressed as means ± standard deviations and statistical analysis was performed with DPS software (Data Processing system) applying one-way ANOVA. Statistical differences among the treatments with *p* < 0.05 was expressed as significant. Graphs were visualized by OriginPro 2022, whereas heatmaps were generated by R programming language using library “pheatmap”.

## 3 Results

### 3.1 Observation of root phenotypes, dry matter accumulation and relative water contents and As contents of germinating soybean seedling

**Figure 1:**
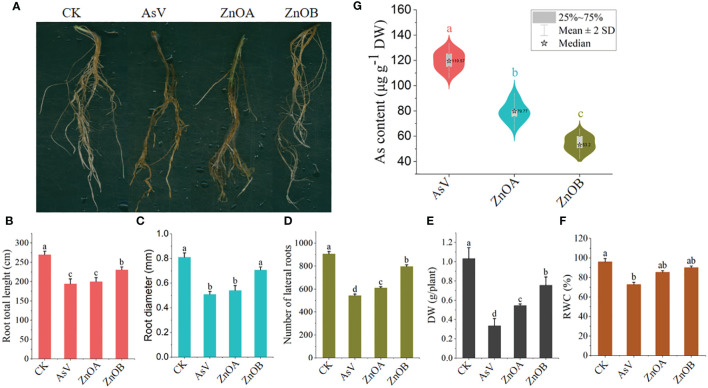
ZnONPs relieve AsV toxicity in soybean roots and inhibit As accumulation. **(A)** Root phenotypes, **(B)** total root lengths, **(C)** root diameters, **(D)** numbers of laterals roots, **(E)** dry weights (DW), **(F)** relative water contents (RWC); and **(G)** As contents of soybean germinating seedling 10 days after AsV and AsV + ZnONPs treatments. The different treatment groups were CK (control), AsV (25 μmol L^−1^ of arsenate), ZnOA (25 μmol L^−1^ of arsenate + 25 μmol L^−1^ of ZnONPs), and ZnOB (25 μmol L^−1^ of arsenate + 50 μmol L^−1^ of ZnONPs). Data are means ± SDs (n = 3). Different lowercase letters indicate significant differences using Tukey’s *post-hoc* test, *p <* 0.05).

The effect of ZnONPs on root morphology, DM and RWC of the As(V)-stressed seedlings are presented in **Figures 1A-F**. The results show that the AsV-only treatment inhibited the growth of the soybean roots by reducing total root lengths, root diameters and lateral and secondary branching. The high-dose ZnONP supplementation mitigated the changes in growth caused by As(V) to a large extent, as evidenced by longer and more vigorous roots than the AsV-only treatment (**Figures 1A-D**). DM and RWC decreased by 67% and 24% in the AsV-only treatment compared to the control plants. Decreases in these parameters in ZnONP-supplemented plants were less than in the AsV-only treatment; DM decreased by 47% and 26% and RWC decreased by 11%, and 6%, respectively, compared to the control treatment when supplemented with 25 μmol L^−1^ and 50 μmol L^−1^ ZnONPs. Supplementation of plants with ZnONPs into the As(V)-containing medium reduced the As contents in roots in a dose dependent manner (**Figure 1G**). There was a 23% decrease in As content in soybean roots under ZnOA treatment and a 55% decrease under ZnOB treatment, both when compared to the As-alone treatment.

### 3.2 Gas exchange parameters, photosynthetic pigment contents and chlorophyll fluorescence

**Figure 2:**
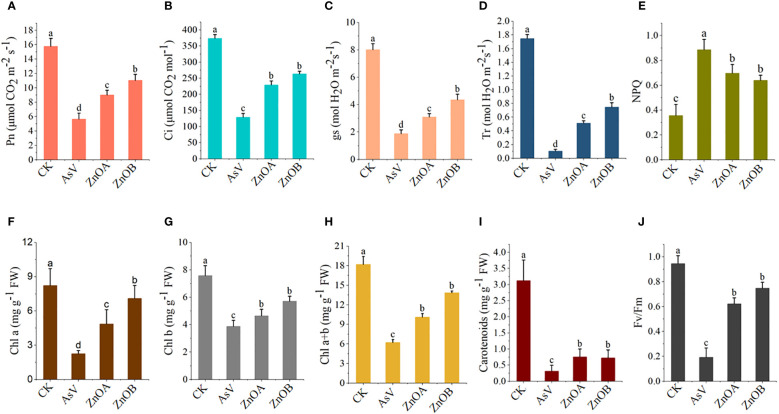
Responses of gas exchange parameter and photosynthetic pigmentation of soybean seedlings. **(A)** Net photosynthesis (*Pn*); **(B)** intercellular CO_2_ concentrations (*Ci*); **(C)** stomatal conductance (*g*); **(D)** transpiration rates (*Tr*); **(E)** non-photochemical quenching (NPQ); **(F)** chlorophyll a (Chl *a*); **(G)** chlorophyll b (Chl *b*); **(H)** Chl *a*+*b*; **(I)** carotenoids; and **(J)** *Fv/Fm* of soybean seedlings 10 days after AsV and AsV + ZnONPs treatments. The different treatment groups were CK (control), AsV (25 μmol L^−1^ of arsenate), ZnOA (25 μmol L^−1^ of arsenate + 25 μmol L^−1^ of ZnONPs), and ZnOB (25 μmol L^−1^ of arsenate + 50 μmol L^−1^ of ZnONPs). Data are means ± SDs (n = 5). Different lowercase letters indicate significant differences using Tukey’s *post-hoc* test, *p <* 0.05).

The effect of As(V) alone and in combination with ZnONPs on photosynthetic parameters of soybean seedlings was investigated in the present study. Compared with the control, the AsV-only treatment reduced all photosynthetic parameters (*Pn*, *E*, *Ci*, and *gs*), and the application of ZnONPs restored the photosynthetic ability of As(V)-stressed plants to some extent (**Figures 2A-D**). For instance, the *Pn* values were 1.6 and 1.9 times, the *E* values were 4.9 and 7.2 times, the *Ci* values were 1.8 and 2 times, and the *gs* values were 1.6 and 2.3 times higher in the ZnOA and ZnOB treatments, respectively, compared to the AsV-only treatment. Treatment with the higher concentration of ZnONPs (ZnOB, 50 µmol L^-1^) had a larger, positive impact on the photosynthesis-related parameters than the low concentration (ZnOA, 25 µmol L^-1^).

Results pertaining to photosynthetic pigments (chlorophyll *a*, chlorophyll *b*, and carotenoids) and to chlorophyll fluorescence (*Fv/Fm* and NPQ) are presented in **Figures 2E-J**. Analysis showed that the contents of these pigments were significantly reduced in the AsV-only treatment compared to the controls; supplementation with ZnONPs mitigated these effects of As(V) in a dose-dependent manner. For instance, the increases in chlorophyll *a*, chlorophyll *b* and carotenoid contents in the ZnOA and ZnOB treatments compared to plants given the AsV-only treatment were 2.2-, 1.2-, 2.4-fold, and 3.1-, 1.5-, 2.3-fold higher, respectively. Similarly, *Fv/Fm* and NPQ were substantially reduced when the plants were given AsV-only treatment and the effects were again mitigated by supplementation with ZnONPs.

### 3.3 Transcriptomic changes in soybean roots and expression validation

To obtain a genome wide view of the transcriptomic changes in soybean roots under As intoxication, alone and in response to supplementation with ZnONPs, four libraries were constructed from plants in the CK, AsV-only, ZnOA, and ZnOB treatments. RNA-seq of the libraries generated 55.3 GB raw reads which, after processing, comprised 54.2 GB clean reads across all libraries and with a base Q30 (the value of Phred>30) value of about 93%. The clean reads were mapped to the soybean reference genome. Only those reads that mapped to the soybean reference genome were processed further; unmatched reads were discarded. About 88 to 92% of clean reads mapped to the reference genome (**Supplementary Figure S1A**). The data pertaining to all samples were then subjected to principal component analysis (PCA) to visualize reproducibility indices (**Supplementary Figure S1B**). The results showed that there was a high degree of reproducibility between replicates and that there were differences in gene expression patterns among in the plants in the different treatments.

Furthermore, the differences in the abundance of DEGs identified by RNA-seq were further confirmed through qRT-PCR validation. From the treatment groups, twelve DEGs associated with stress response, hormonal regulation, and transporter functions were chosen for analysis. Regression analysis exhibited a positive linear correlation between the qRT-PCR and RNA-Seq data results i.e., AsV (R^2 ^= 0.9515), ZnOa (R^2 ^= 0.9667), and ZnOB (R^2 ^= 0.9371) as depicted in **Supplementary Figures S2A-C**. This analysis suggests that the RNA-seq data acquired here are reliable.

### 3.4 ZnONP supplementation modulates the transcriptomic signature of soybean roots by reversing As(V) toxicity

**Figure 3:**
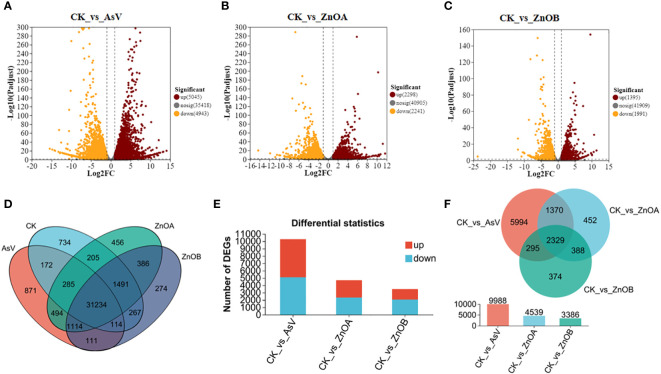
Different transcriptomic expressional patterns of soybean roots after treatment with AsV and ZnONPs. Volcano plots of differentially expressed genes (DEGs) in soybean roots between the following treatment combinations: **(A)** CK_vs_AsV; **(B)** CK_vs_AsV + ZnOA and **(C)** CK_vs_AsV + ZnOB relative to the control. Red dots in the plots represent upregulated DEGs, yellow dots represent downregulated DEGs, and black dots represent unchanged genes. **(D)** Venn diagram of the DEGs in the four treatment groups. **(E)** Barplots showing overall significantly up- and down-regulated DEGs. **(F)** Venn diagram of all significantly up-regulated and down-regulated DEGs among the three treatments groups. The different treatment groups were CK (control), AsV (25 μmol L^−1^ of arsenate), ZnOA (25 μmol L^−1^ of arsenate + 25 μmol L^−1^ of ZnONPs), and ZnOB (25 μmol L^−1^ of arsenate + 50 μmol L^−1^ of ZnONPs).

The expression profiles of the DEGs are presented in volcano plots (**Figures 3A–C**) and venn diagram (**Figure 3D**). Compared with the untreated group, the transcriptomic data of three treated groups suggested that: (i) 5045 genes were up-regulated and 4943 genes were down-regulated after treatment with AsV alone (**Figure 3A**); (ii) 2298 genes were up-regulated and 2241 genes were down-regulated after the ZnOA treatment (**Figure 3B**); and (iii) 3386 genes were differently expressed after the ZnOB treatment, with 1395 genes up-regulated and 1991 genes down-regulated (**Figure 3C**). The numbers of up-regulated DEGs were higher in the AsV-only and ZnOA treatments than were down-regulated, whereas the numbers of down-regulated DEGs were higher in the ZnOB treatment (**Figure 3E**). Furthermore, among the significantly regulated DEGs, 2329 genes were commonly regulated within the three treatment groups, while 5994 DEGs were exclusively regulated in the As-only group and 452 and 374 DEGs were uniquely regulated in ZnOA and ZnOB treatments, respectively (**Figure 3F**). Some DEGs that were expressed in the treatments with As(V) alone and in combination with ZnONPs had significantly different expression trends.

### 3.5 As(V) tolerance due to supplementation with ZnONPs is associated with the mitigation of ROS and induction of stress-responsive genes

**Figure 4:**
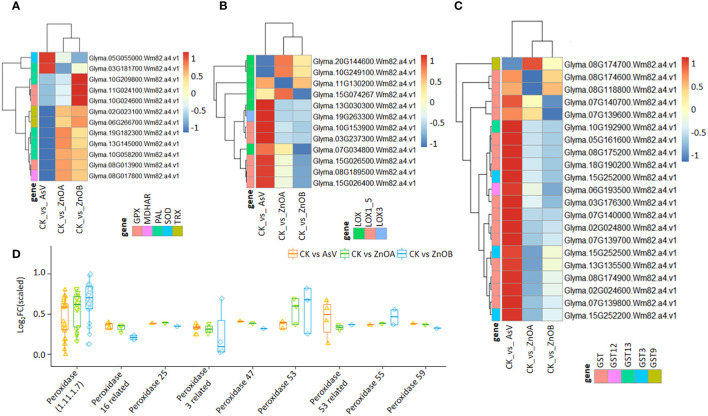
ZnONPs-modulated stress responsive DEGs under As stress in soybean roots. Heatmaps of the expression patterns of DEGs encoding: **(A)** GPX, MAHDR, PAL, SOD, and Trx; **(B)** LOX; and **(C)** GST. **(D)** Expression pattern of DEGs related to peroxidase. The scale represents normalized log2 fold change values. The different treatment groups were CK (control), AsV (25 μmol L^−1^ of arsenate), ZnOA (25 μmol L^−1^ of arsenate + 25 μmol L^−1^ of ZnONPs), and ZnOB (25 μmol L^−1^ of arsenate + 50 μmol L^−1^ of ZnONPs).

To explore the possible role of ZnONPs in the mitigation of As(V) toxicity, we examined the expression patterns of stress-responsive genes. Stress responsive genes putatively encoding peroxidase (*GmPOD*), glutathione S-transferase (*GmGST*), glutathione peroxidase (*GmGPX*), monodehydroascorbate reductase (*Gm*MDHAR), theriodoxin (*GmTrx*), lipoxygenase (*GmLOX*) and phenylalanine ammonia-lyase (*GmPAL*) were found to be differentially regulated among the treatment groups (**Figure 4**; **Supplementary Table S2**). For instance, three DEGs encoding *GmGPX*, two encoding Trx (*Glyma.02G023100.Wm82.a4.v1*, and *Glyma.06G266700.Wm82.a4.v1*), one encoding SOD (*Glyma.05G055000.Wm82.a4.v1*), one encoding MDHAR (*Glyma.08G017800.Wm82.a4.v1*) and four PAL-encoding DEGs showed dynamic expression patterns among the treatments (**Figure 4A**). In particular, the SOD-encoding DEG was only down-regulated in the AsV-only treatment but remained unchanged in plants supplemented with ZnONPs. The expression of 12 DEGs encoding lipoxygenases (*LOX, LOX3* and *LOX1-5*) were differentially modulated in the AsV-only treatment, whereas six genes encoding *LOX* enzymes in the two ZnONP treatments showed diverse expression patterns relative to the control (**Figure 4B**). Most of the GST-encoding DEGs were highly up-regulated by ZnONP supplementation compared to the AsV-only treatment group (**Figure 4C**). *Glyma.08G118800.Wm82.a4.v1*, encoding GST, was 9-fold more up-regulated in response to the ZnONPs supplementation relative to the AsV-only treatment. This suggests that under ZnONPs supplementation, GST-encoding genes play a key role in modulating As(V) tolerance and detoxification. Similarly, the peroxidase-related DEGs were mostly up-regulated in the AsV-only and As(V)+ZnONPs treatments relative to the control. The genes encoding peroxidases were more up-regulated under ZnONPs supplementation than the AsV-only treatment (**Figure 4D**).

### 3.6 ZnONPs-modulate metal transporters in soybean roots under As(V) stress

In this study, expression of 40 genes encoding various transporters was differentially regulated in the AsV-only and As(V)+ZnONPs treatments (**Supplementary Figures S3A, B**; **Supplementary Table S3**). Inorganic phosphate transporters play a major role in the uptake of AsV in plants and among the transporters identified in this study, five inorganic phosphate transporters were induced in the AsV-only treatment, but down-regulated in the ZnONPs group. It was noted that upon ZnONPs supplementation, lower As contents was observed in soybean roots, which can be linked with the reduced abundance of phosphate transporters in roots under ZnONPs supplementation. There were 13 DEGs encoding ZIP family (zinc/iron regulated-like protein) transporters that showed differential expression patterns due to the AsV-only and ZnONPs treatments, of which two ZIP family members, *Glyma.18G078600.Wm82.a4.v1*, and *Glyma.08G328000.Wm82.a4.v1* (both putative homologs of *At*ZIP11), were exclusively up-regulated in the AsV-only treatment. The other DEGs related to this family were mostly down-regulated in the treatments (**Supplementary Figure S3A**). Furthermore, 20 genes encoding ATP-binding cassette (ABC) transporters were differentially regulated in the AsV-only and As(V)+ZnONP treatments (**Supplementary Figure S3B**). Among them, 15 DEGs were up-regulated and five were down-regulated. Notably, the expression of up-regulated ABC transporters was higher in the As(V)+ZnONPs treatment than without ZnONP addition.

### 3.7 Alterations in plant hormone signaling under As stress in response to ZnONPs in soybean roots

**Figure 5:**
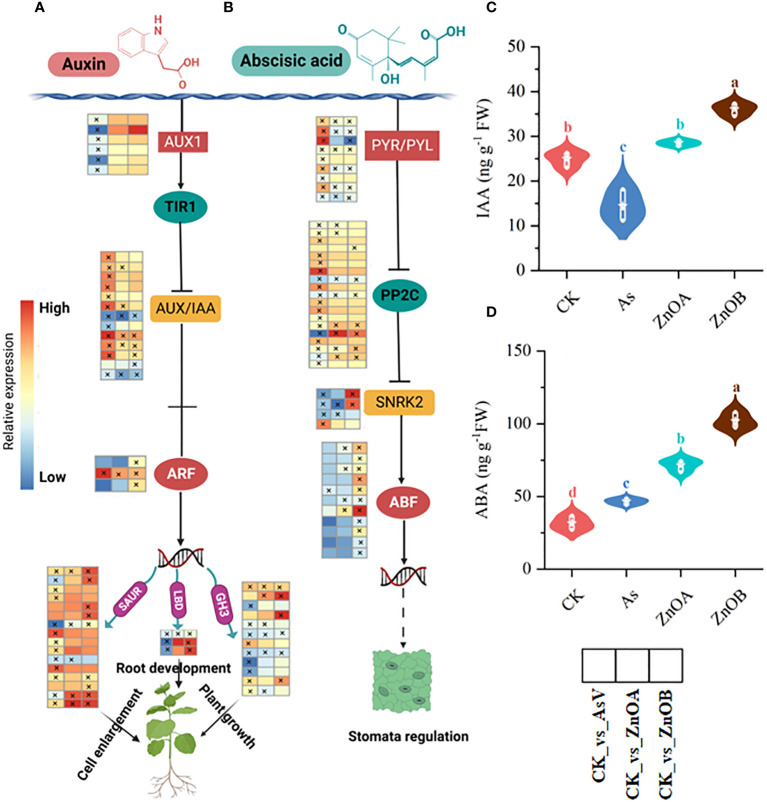
ZnONPs modulate auxin and abscisic acid signaling pathways under AsV intoxication. **(A)** Auxin signaling pathway. **(B)** Abscisic acid signaling pathway. **(C)** Indole acetic acid content (IAA). **(D)** Abscisic acid content (ABA). Heatmaps were generated from log2 fold change values of DEGs of respective treatment groups relative to controls and the asterisks in the heatmaps represent the DEGs in that specific treatment. The different treatment groups were CK (control), AsV (25 μmol L^−1^ of arsenate), ZnOA (25 μmol L^−1^ of arsenate + 25 μmol L^−1^ of ZnONPs), and ZnOB (25 μmol L^−1^ of arsenate + 50 μmol L^−1^ of ZnONPs). Results in the violin plots represent the means ± SDs (n = 3). Different lowercase letters represent significant differences Tukey’s *post-hoc* test, *p ≤* 0.05.

Our data showed that 44, 19 and 27 DEGs related to the auxin signaling pathway (such as auxin transporter like-proteins/auxin influx carriers (*AUX1), AUX/IAA*, auxin response factor *(ARF), Small auxin up RNA (SUAR), Gretchen hagen (GH3)*, and *lateral organ boundaries domain (LBD*) were differentially regulated in the AsV-only, ZnOA and ZnOB treatments, respectively (**Figure 5A**; **Supplementary Table S4**). Upon imposition of As(V) stress, six AUX1 were down-regulated whereas their expression remained unchanged upon supplementation with ZnONPs. Similarly, most of the DEGs encoding AUX/IAA proteins, which are involved in auxin signal transduction, were induced in the AsV-only treatment whereas, with supplementation with ZnONP, only four and three DEGs were induced and two and three DEGs were suppressed, respectively, in the ZnOA and ZnOB treatments. Furthermore, only one gene encoding an auxin transcription factor (ARF) was induced in the AsV-only treatment, while four genes of this type were induced in the plants supplemented with ZnONPs. Other genes downstream of ARF involved in the auxin signaling pathway such as *SAUR* and *3GH3* were down-regulated due to As(V). In contrast, these genes were up-regulated or showed no changes in expression in the treatments supplemented with ZnONPs. Surprisingly, two *LBD* genes, which play a crucial role in stress tolerance and plant architecture, were up-regulated only in response to ZnONP supplementation. To further confirm the responses of the auxin signaling pathway, we determined the content of IAA in root tissues and found that IAA contents increased compared to the control by the addition of ZnONPs and reduced below concentrations in the controls by As(V) (**Figure 5C**). These results suggest that auxin signaling pathways are involved in the mitigation of As(V) stress by ZnONPs.

Abscisic acid (ABA) is a core signaling molecule modulating plant growth and development under both non-stressed (Raghavendra et al., 2010) and stress conditions (Denancé et al., 2013; Zvobgo et al., 2018b). The core components of the ABA signaling pathway are pyrabatin resistance/pyrabatin resistance 1-lik*e* (PYR/PYL) ABA receptors, protein phosphate 2C (PP2C), sucrose nonfermenting-1-related protein kinase 2 (SnRK2s), and *ABSCISIC ACID-INSENSITIVE* (*ABI*). In this dataset, in response to the AsV-only treatment, we found that the expression of PYL/PYR-encoding genes were induced whereas the expression of PP2C*-*encoding genes was suppressed resulting in down-regulation of the SnRK2s family. In contrast, in response to treatment with ZnONPs, PYL/PYR-encoding DEGs were down-regulated resulting in the up-regulation of PP2C and SnRK2s and, subsequently, induced downstream ABF transcription factors (**Figure 5B**). ABA concentrations were lowest in the control plants, slightly raised in the AsV-only treatment but increased substantially by supplementation with ZnONPs (**Figure 5D**). Taken together, this indicates that ABA signaling and response are modulated in As(V)-stressed roots in response to the ZnONPs.

**Figure 6:**
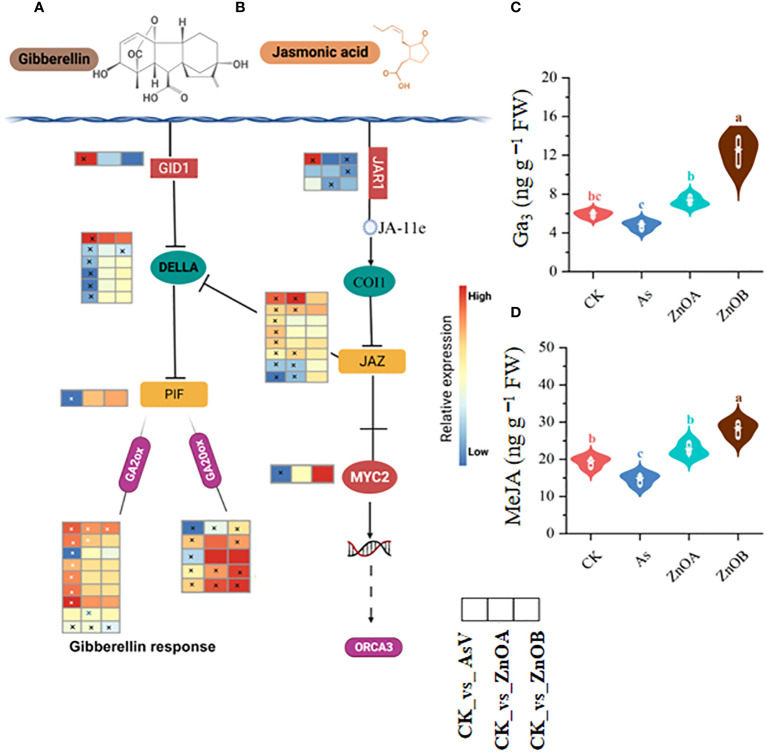
ZnONPs modulate the gibberellin and jasmonic acid signaling pathways under AsV intoxication. **(A)** Gibberellin signaling pathway. **(B)** Jasmonic acid signaling pathway. **(C)** Gibberellic acid contents (GA_3_). **(D)** Methyl jasmonate contents (MeJA). Heatmaps were generated from log2 fold change value of DEGs of respective treatment group relative to controls and the asterisks in the heatmaps represent the DEGs in that specific treatment. The different treatment groups were CK (control), AsV (25 μmol L^−1^ of arsenate), ZnOA (25 μmol L^−1^ of arsenate + 25 μmol L^−1^ of ZnONPs), and ZnOB (25 μmol L^−1^ of arsenate + 50 μmol L^−1^ of ZnONPs). Results in the violin plots represent the means ± SDs (n = 3). Different lowercase letters represent significant differences using Tukey’s *post-hoc* test, *p ≤* 0.05.

The GA signaling pathway was also significantly modulated by the AsV-only, ZnOA and ZnOB treatments (**Figure 6A**). For instance, DEGs involved in gibberellin signaling pathways such as *GIBBERELLIN INSENSITIVE DWARF1 GID1B* (*Glyma.03G148300.Wm82.a4.v1*), a gibberellin receptor, were significantly up-regulated, whereas most of the DELLA transcripts, which is a gibberellin repressor, and one phytochrome-interacting factor 3 (PIF3) transcription factor were down-regulated in the AsV-only treatment. None of these genes were differentially expressed under ZnONPs supplementation except for *Glyma.06G213100.Wm82.a4.v*1 that encodes a DELLA protein. Other gibberellin signaling-related genes such as *gibberellin 2 oxidase* (*GA2ox*), which catalyzes the degradation of GA, was up-regulated in response to the AsV-only treatment; however, it showed diverse expression patterns in the ZnONPs treatments. It is important to note that the expression of most of the *gibberellin 20 oxidase* (*GA20ox*) genes remained unchanged in response to ZnONPs, suggesting that ZnONPs treatment relieved the As(V) stress and promoted the soybean growth without regulating GA-related genes. We also found higher GA_3_ contents in soybean roots in response to ZnONPs treatment than in the control and AsV-only treatments (**Figure 6C**).

In the jasmonic acid signaling pathway, As(V) induced the expression of one gene encoding jasmonic acid-amido synthetase (*JAR1*), four TIFY proteins of the JAZ subfamily, but down-regulated two *JAR1-*encoding genes, one *MYC2* TF-related gene, and two *JAZ-*encoding genes. None of the *JAR1* encoding gene was up-regulated due to ZnONPs treatment, but we identified three TIFY proteins of the JAZ subfamily whose expression were up-regulated in response to the ZnOA treatment (**Figure 6B**). In addition, we analyzed MeJA contents in soybean roots and found that they were increased in ZnONP-treated samples compared to the controls and the AsV-only plants. There were 1.5- and 1.9-fold increases in the ZnOA and ZnOB treatments over the AsV-only treatment (**Figure 6D**). These results collectively suggested that ZnONP supplementation modulated phytohormone biosynthesis and signaling pathways, thereby mitigating the effects As(V) toxicity.

### 3.8 Co-expression network analysis reveals modules with different expression patterns and their association with physiological traits

**Figure 7:**
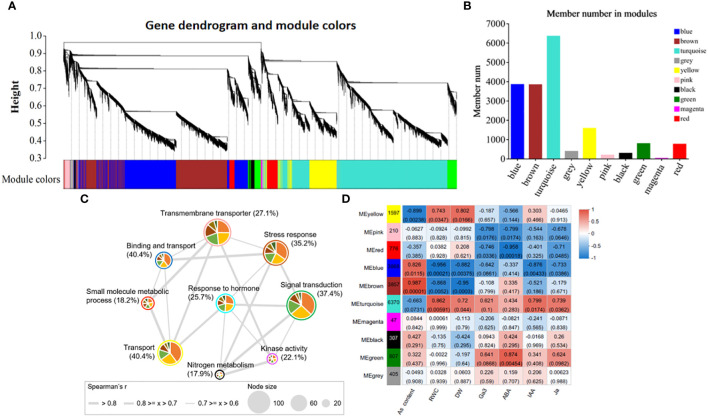
Weighted gene co-expression network analysis and correlation of trait-module analysis. **(A)** Clustering dendrogram tree showing co-expression modules. **(B)** The number of members in each module, **(C)** Interacted network-analysis of significantly enriched gene ontology (GO) terms among the 9 plant gene modules. **(D)** Physiological trait-module correlation analysis. The different colors circled around each node correspond to specific module and node size represents to the number of GO terms interacting in that specific module. The thickness of the edges represents the correlation strength.

Weighted gene co-expression network analysis (WGCNA) can provide an effective means of revealing the molecular mechanism of As(V) tolerance in response to ZnONPs by identifying key gene modules. The clusters (modules) were identified from the dendrogram in **Figure 7A** as each tree branch represents a module and each leaf in a branch represent a gene. The dendrogram analysis classified the DEGs into a total of 10 modules (differentiated by color). The number of genes in each module is shown in **Figure 7B**. The highest number of DEGs (6370) were in the MEturquoise module followed by 3868 genes in the MEblue module; the lowest number of DEGs (47) were found in the MEmagenta module. Furthermore, the correlation of modules with the physiological traits revealed that five modules (MEpink, MEblue, MEbrown, MEturquoise and MEgreen) were significantly correlated with physiological traits (**Figure 7D**). Among these modules, MEblue and MEbrown have the highest, positive correlation with As contents. The MEturquoise and MEgreen modules have positive correlations with plant hormones, while the MEpink module correlates with RWC and DW. Moreover, through the integration of the identified modules with GO terms, we detected nine plant gene modules that formed an interactive network (*r* > 0.6, *p* < 0.05; **Figure 7C**). These gene modules represent different GO functions, e.g., signal transduction (MEgreen module), transmembrane transporter (MEpink module), stress response (MEbrown module), response to hormones (MEturquoise module).

### 3.9 Identification of hub genes in targeted modules

**Figure 8:**
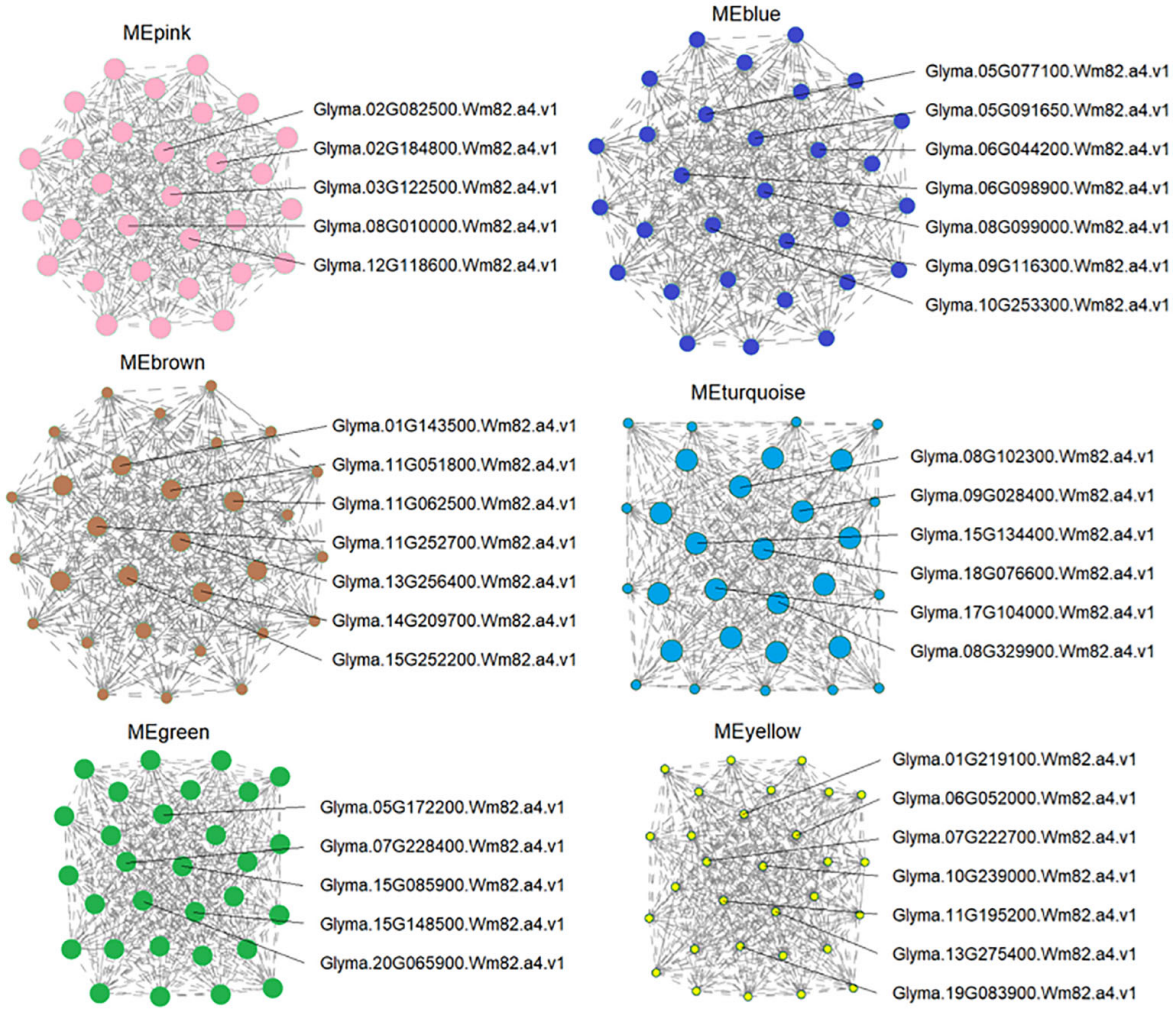
Candidate hub gene expression identified by co-expression networks. Candidate hub genes were selected based on those modules shown high correlation with physiological traits. The whole network of hub genes of each module was enlarged, and the hubs and surrounding nodes and edges were screen-captured and shown.

To identify the possible key regulators of As tolerance in response to ZnONPs supplementation, we found 37 major expression hub genes that showed a strong association with DEGs in targeted modules (**Figure 8**; **Supplementary Table S5**). Several of these hub genes are related to stress tolerance, transporters, phytohormone signaling, cellular signaling and cell death. For example, in the MEpink module, five hub genes were identified, of which the *NITRATE TRANSPORTER 1/PEPTIDE TRANSPORTER* (*NRT1/PTR*) homolog, *Glyma.03G122500.Wm82.a4.v1*, showed modulated regulation due to the AsV-only and As(V)+ZnONPs treatments. *PTR* genes are essential transporters for many substrates in plants, including nitrate, secondary metabolites, peptides, and hormones (Wen et al., 2020). *Glyma.02G082500.Wm82.a4.v1* encodes *vacuolar iron transporter homolog 3* (*VIT*3) and remained unchanged in the AsV-only and ZnOA treatments but was down-regulated in the ZnOB treatments. Another transcript, *Glyma.08G010000.Wm82.a4.v1*), of the MEpink module is related to trans-membrane transporter activity was down-regulated under ZnOB treatment.

Module MEyellow contains seven hub genes that are involved in transport and transmembrane transporter activity; all these genes showed diverse expression patterns due to the different treatments. Of these, two genes, *Glyma.11G195200.Wm82.a4.v1* and *Glyma.07G222700.Wm82.a4.v1*, belong to the MFS_1 (Major Facilitator Superfamily) superfamily and are associated with sugar transporters and high-affinity nitrate transporters. Two genes of the MATE/SLC47A family, *Glyma.10G239000.Wm82.a4.v1* (protein DETOXIFICATION 49) and *Glyma.13G275400.Wm82.a4.v1* (protein DETOXIFICATION 19), showed reduced expression under all treatment combinations. This family is a secondary transport family mainly responsible for heavy metal/toxic compound detoxification (Wang et al., 2022). Three other homologs in this module with differential expression patterns are Cu^+^-exporting ATPase (*Glyma.01G219100.Wm82.a4.v1*), a zinc transporter (ZIP) (*Glyma.06G052000.Wm82.a4.v1*), and an amino acid transporter (*Glyma.19G083900.Wm82.a4.v1*).

The annotation of the seven hub genes in MEblue showed they are related to kinase-like proteins (*Glyma.10G253300.Wm82.a4.v1*, *Glyma.06G098900.Wm82.a4.v1*, *Glyma.09G116300.Wm82.a4.v1*), *NRAMP2* (*Glyma.06G044200.Wm82.a4.v1*), the chloride channel protein, CLC-b (G*lyma.05G077100.Wm82.a4.v1*), transitional endoplasmic reticulum ATPase (*Glyma.05G091650.Wm82.a4.v1*), and hypothetical protein (*Glyma.08G099000.Wm82.a4.v1*). The regulation of hub genes of the three modules (MEpink, MEyellow, and MEblue) collectively suggest their participation in heavy metal transport and detoxification in soybeans.

Two hub genes in MEturquoise have functions related to plant hormones and signal development such as *Patellin-4* (*Glyma.09G028400.Wm82.a4.v1*, *Glyma.15G134400.Wm82.a4.v1*) and are associated with diverse signaling pathways, such as response to cytokinins (Černý et al., 2011) and auxins (Tejos et al., 2017). Their expression was suppressed in the AsV-only treatment while showing no change in expression in response to the application of ZnONPs. Furthermore, two candidate hub genes encoding *fasciclin-like arabinogalactan protein 1* (*FLAs*; *Glyma.08G329900.Wm82.a4.v1* and *Glyma.18G076600.Wm82.a4.v1*) were also do wn-regulated in the AsV-only treatment, but their expression was unchanged by ZnONPs supplementation.

## 4 Discussion

Soybean is a nutritionally rich crop but, unfortunately, it is often cultivated on marginal and arsenic-contaminated land in the southern provinces of China causing crops grown in this region to have poor yields and quality. Genomic resources for this important leguminous crop are lacking. Therefore, exploring the molecular mechanisms associated with arsenic tolerance is crucial for facilitating breeding soybean cultivars with high arsenic tolerance. The authors believe that this is the first study evaluating transcriptomic signatures in soybean roots after being subjected to As(V) stress with or without supplementation with ZnONPs.

### 4.1 ROS mitigation and regulation of stress responsive genes are associated with As(V) tolerance in response to ZnONP supplementation in soybean roots

Stress due to arsenic intoxication induces the accumulation of ROS that inhibit chlorophyll production and limit photosynthetic activity resulting in reduced growth (Thakur et al., 2019; Zeeshan et al., 2022). Increased production of ROS causes oxidative stress which reduces the integrity of cell membranes and causes changes to proteins, lipids, and DNA (Thakur et al., 2019; Zeeshan et al., 2022). Our data set showed that As(V) stress-induced *LOX2S* and *LOX1-5* in soybean roots, whereas simultaneous treatment with ZnONPs suppressed the expression of these genes in a dose-dependent manner. *LOX* genes have a role in catalyzing the peroxidation of unsaturated fatty acids of bio-membranes resulting in the generation of hydroperoxides and active oxygen species (You et al., 2011). Previously, we have found that amendment with ZnONPs and SeNPs, either alone or in combination, relieved oxidative stress in As(V)-treated soybean tissues (Zeeshan et al., 2021, 2022), results that can be explained by the low expression of *LOX* genes found in this study. Taken together, the responses to ZnONPs maybe through reduced oxidative stress resulting in maintaining the biosynthesis of photosynthetic pigments, water balance, and chloroplast structure.

To deal with oxidative stress, plants develop protective mechanisms to remove ROS. In this study, the RNA-seq data demonstrated that genes encoding GST, POD, GPX, Trx, SOD and MDHAR showed increased expression due to ZnONP-supplementation, and one GST-encoding gene (*Glyma.15G252200.Wm82.a4.v1*) is among the candidate hub genes in MEbrown module. Previously, we found that ZnONP-supplementation increased the expression of *GmSOD* and *GmCAT* genes whose functions are involved in the scavenging of ROS (Zeeshan et al., 2021, 2022).Plant GSTs have a major role in detoxifying hydroperoxides and xenobiotics and protect cells from lipid peroxidation (You et al., 2011). In this process, SOD catalyzes the conversion of O_2_ **^·−^** to H_2_O_2_, which is then to converted to O_2_ and water molecules by the action of CAT. Similarly, GPX and POD also play active roles in the removal of H_2_O_2_ by reducing it to oxygen (Herbette et al., 2007; Passaia et al., 2014). Thioredoxin (Trx) also has role in modulating hormone signaling, the production transcription factors, and DNA synthesis to protect cells from toxicants (Ouyang et al., 2018; Zhang H. et al., 2018). Taken together, these enzymes are key components of the plant defense system and through their coordination provide protection to cells against As(V) stress.

### 4.2 Dynamic expression of transporters in response to ZnONPs contribute to As(V) tolerance

Changes in arsenic translocation and sequestration into less sensitive cellular organs (such as vacuoles) are well-known tolerance mechanisms in plants (Zeeshan et al., 2023). Studies have shown that As(V) is taken up and transported by phosphate transporters (PHTs) in the roots (Zvobgo et al., 2018a; Zeeshan et al., 2022). In the current study, we identified five *inorganic phosphate transporter* (*PHTs*) genes, of which *Glyma.07G222700.Wm82.a4.v1* is a hub gene of the MEyellow module. The expression of these genes was induced upon As(V) stress; supplementation with ZnONPs reduced their expression. Phosphate transporters have either a low or high affinity and are responsible for As(V) uptake because of the similarity in chemical structure of arsenic with phosphate (Jian et al., 2008). Studies have found that two phosphate transporters were less induced in arsenic-tolerant genotypes than in sensitive ones during P starvation (Puckett et al., 2012; Zvobgo et al., 2018b) suggesting that P starvation promotes the uptake of As(V) via Pi transporters. Likewise, the over-expression of two soybean Pi transporters, *GmPT1* and *GmPT4*, enhanced As(V) uptake in *Arabidopsis* (Shin et al., 2004). This is consistent with higher concentrations of arsenic in soybean roots in the AsV-only treatment than in the other treatments. It is interesting to note that, in the current study, treatment with ZnONPs reduced the expression of Pi transporter genes in soybean roots. It is known that Zn deficiency in the root medium causes a loss of control transcription of Pi transporter genes in barley (Huang et al., 2000) and *Arabidopsis* (Jain et al., 2013; Khan et al., 2014) leading to accumulation of Pi, and Huang et al. (2000) suggest that Zn ions have a specific role in the regulation of genes encoding P transporters in plant roots. Supplementation with ZnONPs may, therefore, help control the transcription of PHT transporters reducing their expression and resulting in lower As(V) uptake; however, this needs further elucidation.

Zinc is the second largest trace element after iron and plays an important role in the modulation of different physiological and molecular processes and, in plants, its uptake is regulated by zinc transporters, particularly ZIP family proteins (Amini et al., 2022; Maharajan et al., 2023). ZIP family proteins are not only an important component of the Zn^2+^ uptake and transport system but are also involved in the uptake of other divalent metals such as cobalt, cadmium, copper, iron, and manganese (Pedas et al., 2009).In this study, most of *Gm*ZIP family members were down-regulated in the three treatments except for *Glyma.18G078600.Wm82.a4.v1* and *Glyma.08G328000.Wm82.a4.v1* that were up-regulated, compared to the controls, of which, one gene (*Glyma.06G052000.Wm82.a4.v1*) was identified as a hub gene in the MEyellow module. Previously, *At*ZIP family genes have shown reduced expression levels in roots and shoots of *Arabidopsis thaliana* under excessive supplementation with Zn^2+^ and ZnONPs (Nair and Chung, 2017). These authors attributed the reduced expression of these DEGs to Zn homeostasis via low uptake. Similarly, *ZIP-*encoding genes were also suppressed under excessive Zn^2+^ applications in various crop species (van de Mortel et al., 2006; Jain et al., 2013). In addition, three ZIP family proteins (*Hv*ZIP3, *Hv*ZIP5, and *Hv*ZIP8) showed high expression under Zn^2+^-deficient conditions in barley (Pedas et al., 2009). Similarly, several ZIP transporters in *Arabidopsis* were also induced under Zn^2+^ depleted condition and reduced their expression when the plant were transferred to Zn^2+^ normal conditions (van de Mortel et al., 2006). In the current study, the reduced As(V) contents of the plants supplemented with ZnONPs may be due to the reduced expression of most of the genes encoding *ZIPs*.

The ABC transporter proteins play an important role as channels for the uptake of essential nutrients and toxic elements into plants (Kang et al., 2011; Zvobgo et al., 2018b). After uptake, As(V) is reduced into As(III) by the action of the enzyme, arsenate reductase, which then complexes with thiol compounds such as phytochelatins (PCs) and GSH (Kumar and Trivedi, 2018) and is transported to less sensitive cell organs by ABC transporter family proteins (Song et al., 2014; Kumar and Trivedi, 2018). In this study, we identified 21 ABC-type transporter genes belonging to the A, B, C and G subfamilies that were all up-regulated in all treatments; however, their expression was relatively higher in the As(V)+ZnONPs treatments than in the AsV-only treatment. The increased expression of ABC transporters due to ZnONP supplementation might be because these transporters are not only involved in the detoxification process (as mentioned earlier) but also facilitate the uptake of essential nutrients (Zvobgo et al., 2018b). In addition, in our previous study, supplementation of ZnONPs increased the production of PC contents in roots and shoots of As(V)-stressed soybean plants, and high accumulation of arsenic contents in vacuoles, suggesting its role in As(V) detoxification (Zeeshan et al., 2022). The lower accumulation of As in soybean roots in response to the ZnONPs treatments suggests a role for these transporters in As(V) tolerance.

### 4.3 Hormonal interplay in the presence of ZnONPs involves in As(V) tolerance

As sessile organisms, plants must evolve physiological and developmental adaptations to combat unfavorable conditions and use signaling molecules and mechanisms that mediate (re)patterning at the tissue and cellular level to adapt to the prevailing conditions. Phytohormones are involved in many aspects of plant development and are responsible for intra- and inter-cellular communication and modulation of cellular processes (Tejos et al., 2017). Our analyses showed that the AsV-only treatment and supplementation with ZnONPs modulated several hormone-related genes such as *GH3, AUX/IAA*, *LBD*, and *SAUR*. *AUX/IAA* is a negative regulator of auxin transduction by suppressing the ARF transcription factor (Liu et al., 2020), whereas *SAUR* acts as a positive regulator of the auxin signaling pathway (Stortenbeker and Bemer, 2019). In *Arabidopsis*, high concentrations of auxin suppress the AUX/IAA protein and increase transcription of ARF which directly regulates the expression of *LBD* family genes (Goh et al., 2012). Auxin response factor 3 (*ARF3*) was specifically up-regulated in the ZnOB treatment, whereas, it remains unchanged in the AsV-only and ZnOA treatments, indicating its involvement in the modulation of the auxin signaling of As(V)-stressed soybean roots supplemented with a high dose of ZnONPs. Three genes of the *LBD* family, which encode auxin-responsive *lateral organ boundaries* (*LOB*) gene and are responsible for lateral roots development (Majer and Hochholdinger, 2011) were downregulated in the AsV-only treatment but remained unchanged in the As(V)+ZnONPs treatments, suggesting that ZnONP supplementation relieved the As(V) stress by promoting the lateral root development as evidenced by improved root architecture observed during this study (**Figures 1A–D**).

This study identified several DEGs encoding the three principal components of the ABA signaling pathway, PYR/PYL, PP2C and SnRK2s, in the AsV-only and As(V)+ZnONPs treatment groups. PYR/PYL proteins are ABA receptors that upon activation release the PP2C protein which in turn regulates SnRK2s and activation of downstream targets (Raghavendra et al., 2010; Denancé et al., 2013). In the presence of ABA, the PYR/PYL complex tightly links with PP2C, thereby inhibiting PP2C-mediated dephosphorylation of SnRK2. This, in turn, allows activated SnRK2s to relay the ABA signal (Manohar et al., 2017). In a previous study, the expression of genes of the ABA signaling pathway module PYR/PYL-PP2C-SnRK2s were differentially genes due to treatments As(V)-stressed plants either in the presence or absence of an addition of P in the roots of an As-tolerant genotype *Hordeum vulgare*, showing this module plays a role in As tolerance (Zvobgo et al., 2018b). The results of this study confirm this conclusion, as we found higher ABA contents in soybean roots treated with ZnONPs and a smaller number of DEGs of the ABA signaling pathway in plants given the ZnOB treatment.

Jasmonic acid (JA) is lipid-derived signaling hormone and is involved in protecting plants against (a)biotic stresses (Zhang P. et al., 2018). The core components of the JA signaling pathway, such as TIFY10 of the JAZ subfamily and the transcription factor MYC2, were, respectively, up- and down-regulated in the AsV-only treatment. In contrast, the expression of JAZ subfamily genes was altered in the ZnONB treatment. The induction of JAZ family genes represses MYC2 which results in the suppression of JA-responsive gene transcription (Yang et al., 2012). However, it was found that a JAZ protein was suppressed and *MYC2* was induced in tolerant and sensitive genotypes of *Hordeum vulgare* upon imposition of As(V) stress (Zvobgo et al., 2018b), suggesting JA signaling is a complex process that shows variable responses in different crop species. This might be because JA exhibits synergistic and antagonistic crosstalk with auxin, ethylene (Wasternack, 2007), and especially with GA (Yang et al., 2012). It was previously noted that JA signaling was inhibited by the antagonistic effects of GA through JAZ-DELLA interactions and/or DELLA-JAZ interactions (Hou et al., 2010; Yang et al., 2012). In this study, we found low expression of the DELLA protein in response to the AsV-only treatment, whereas its expression was unaltered under ZnONPs supplementation. It seems that the induction of JAZ not only suppressed the MYC*2* TF in AsV-only treatment but also inhibited the DELLA protein through its antagonist crosstalk. Also, there was a high concentration of GA in the treatment groups relative to the control; however, we only found a high expression level of GID1B (GA receptor) in the AsV-only treatment. In the presence GA, a GID1B makes a bond with GA, which facilitates the interaction with the DELLA protein (Achard and Genschik, 2009) resulting in the suppression/degradation of the DELLA protein. In addition, GA2-oxidase, a catabolic enzyme usually activated under stress conditions, reduces bioactive GA level and suppress plant growth and was found to be upregulated in the AsV-only treatment, whereas supplementation with ZnONPs reduced its expression level. Also, the GA synthesis gene, *GA20ox*, was down-regulated in the ZnONP treatments compared with AsV-only treatment. Therefore, these results suggest that GA signaling may be involved in As(V)-induced repression of soybean root growth. Further study is needed to explore the specific role of hormonal interplay in soybean under the concurrent application of As(V) and ZnONPs.

### 4.4 Co-expression network analysis and identification of hub genes by WGCNA showed diverse expression patterns of modules in soybean roots

WGCNA is a progressive data mining approach in which DEGs are divided into different co-expression modules. Genes in each module/cluster are highly interconnected and have similar expression patterns and performing similar physiological functions (Ye et al., 2020). Each module is then checked for its correlation with physiological traits and endogenous hormones. In this study, the DEGs were classified into 10 modules (as shown by different colors) with hierarchical clustering based on an unsigned co-expression network (**Figure 7**). GO analysis showed that DEGs in these modules were highly enriched in biological processes such as response to stress, signal transduction, trans-membrane transport, and response to hormones. To further narrow the range of arsenic tolerance genes related to these biological processes, we identified the hub genes in the five most significant modules having the highest correlation among the modules. We identified 37 principal hub genes in order to determine candidate genes responsible for arsenic tolerance that were regulated by ZnONPs.

Transport-related DEGs are crucial factors of As(V) stress tolerance. Several hub genes encoding transporters showed dynamic expression in the MEPink, MEyellow, and MEblue modules. Of these, NRT1/PTR 3.1 (NPF), a dual-affinity nitrate transporter, was suppressed only in response to the ZnOB treatment. Recently, studies also revealed its role in the transport of ABA, auxin and GA (Chiba et al., 2015). Another hub gene, an uncharacterized membrane protein (*Glyma.02G082500.Wm82.a4.v1*) and a homolog of *Arabidopsis vacuolar iron transporter* (*VIT*), plays a dominant role in iron transport and detoxification in protists, fungi and plant (Slavic et al., 2016). Study revealed that *VIT* also plays a significant role in nitrogen fixation in soybean, as this gene is also homologous to *Lotus japonicus* SEN1 (*LjSEN1*) (Brear et al., 2020). However, given this study, its specific role needs to be revaluated. Another gene, *BIDIRECTIONAL SUGAR TRANSPORTER SWEET10* (*Glyma.08G010000.Wm82.a4.v1*), also known as sugar/sucrose efflux transporter was induced in plants given the ZnOB treatment whereas its expression remained unchanged in the other treatments. The Sugars Will Eventually be Exported Transporter (SWEET) proteins play crucial roles in plant development by translocating sugars from one cell in more distant transport between organs (Wang et al., 2020). Sucrose is the main carbon energy source in plants. The sugars derived from sucrose metabolism provide tolerance against abiotic stresses (Misra and Mall, 2021), and the high expression of the *SWEET* gene positively contributes to sugar accumulation (Wei et al., 2014). Knockout of *GmSWEET10* reduced the oil content and seed size in soybean (Wang et al., 2020).The gene *NRAMP2* (*Glyma.06G044200.Wm82.a4.v1*), is a hub gene in the MEblue module and was induced in the AsV-only treatment. Initial work on the NRAMP gene family suggested that they have a role in Fe uptake, since then they have been linked with the uptake of several other metals (Tiwari et al., 2014 and the references therein) and they have subsequently been shown have potential roles in metal(loid) tolerance by sequestering ions in tolerant cell organs (Ma et al., 2021). The study by Tiwari et al. (2014) also suggested that the overexpression of the *OsNRAMP* gene in a yeast mutant and *Arabidopsis* roots affected As and Cd uptake. The *HvNRAMP5* gene was down-regulated by treatments with As(V) and As(V)+P in barley roots, suggesting its ambiguous role under As(V) stress (Zvobgo et al., 2018b). These genes may play cross-functional roles in tolerance to As(V) stress in response to the ZnONPs supplementation in soybean roots.

Two hub genes encoding *protein DETOXIFICATION 49* and *protein DETOXIFICATION 19* of the MATE family in MEyellow module showed reduced expression in the AsV-only and As(V)+ZnONP treatments. The MATE protein family plays an important role in plant development by modulating plant hormones and providing tolerance against (a)biotic stresses by scavenging toxic substances and secondary metabolites (Li et al., 2002). Generally, in plant cells, MATE proteins are localized in plasma membrane thereby facilitating the efflux of toxic substance from the cytoplasm, and the *Arabidopsis protein DETOXIFICATION 1* gene has been shown to be involved in the efflux of Cd^2+^ from the cytoplasm (Li et al., 2002). Also, in *Arabidopsis*, protein DETOXIFICATION 19 was expressed in root epidermal cells thereby protecting roots from hazardous compounds in the soil (Zheng et al., 2023). Others DETOXIFICATION genes of MATE family also play substantial role such as *AtDTX30* promote aluminum tolerance and regulate root hair roots (Ali et al., 2021). In this study, the downregulation of these genes suggest that they may not play significant role in As(V) tolerance and that soybean has developed a different strategy to detoxify/efflux the As(V) from cell in the presence of ZnONPs.

Two *PATELLINS* (*PATL*) genes are candidate hub genes in the MEturquoise module. In *Arabidopsis*, this protein is associated with plasma membrane mainly particularly in lateral roots, primary roots, embryos, and developing stomata (Tejos et al., 2017) and its function is mainly associated with various phytohormone signaling responses (Černý et al., 2011). qRT-PCR, found that four *PATL* genes showed diverse expression patterns in response to the IAA treatment suggesting that *PATL* genes might be involved in auxin signaling (Tejos et al., 2017). The current study suggests the same maybe the case and that high auxin accumulation and auxin signaling under ZnONPs supplementation might have regulated the two *PATL4* genes.

Two hub genes encoding fasciclin-like arabinogalactan-proteins (FLAs), were identified in the MEturquoise module and were down-regulated in the AsV-only treatment. Previous studies inferred that *FLAs* genes are expressed in various plant tissues such as roots, leaves, stems and flowers and play a crucial function in plant development as well as in adaptation (Deng et al., 2022). For instance, the *ZeFLA11* gene and its homolog in *Arabidopsis* induces secondary cell wall thickening (Dahiya et al., 2006), *AtFLA18* promotes roots elongation (Allelign Ashagre et al., 2021) whereas *OsFLA1* is expressed in anthers and promotes pollen development in rice (Deng et al., 2022). In our previous study, we found that As stress strongly induced ROS accumulation causing lipid peroxidation resulting in cell wall disruption and cell death (Zeeshan et al., 2021). Therefore, the downregulation of two *FLAs* genes under As(V) stress indicates that As(V) stress negatively affects cell wall formation in soybeans. Surprisingly, the *GmPATL* and *GmFLA* genes showed less response to ZnONPs and suggests less need for these genes to contribute in As(V) tolerance in soybean germinating seedling.

## 5 Conclusions

This study was designed to elucidate the physiological changes, hormonal regulation and differential expression of the transcriptome in response to ZnONP supplementation in soybean roots under As(V) stress. Our datasets showed that supplementation with ZnONPs increased photosynthesis efficiency, induced the stress responsive genes, *GmPOD, GmGST, GmTrx, GmGPX* and *GmPAL*, and reduced the oxidative stress generated by As(V) thereby promoting plant growth. ZnONPs also ameliorated As(V) stress by limiting its uptake and facilitated its sequestration as evidenced by down-regulation of *PHT* and *NRAMP* genes and up-regulation of ABC transporters. Furthermore, high contents of phytohormones (IAA, GA_3_, MeJA, and ABA) and the differential expression genes related to the signaling pathways of these hormones were found in response to ZnONP supplementation. Importantly, although this study found DEGs that were common to the ZnONP and AsV-only treatments, the number of DEGs were lower under ZnONP supplementation than in AsV-only treatment. Although this study has given new insights into the mechanisms of As(V) tolerance in soybeans, it will be necessary to functional characterization these DEGs to clarify their roles in biological pathways involved in As(V) metabolism in soybeans.

## Data availability statement

The original contributions presented in the study are publicly available. The data presented in the study are deposited in the Sequence Read Archive (SRA) repository, accession number PRJNA1120636.

## Author contributions

MZ: Conceptualization, Data curation, Formal analysis, Methodology, Writing – original draft. CS: Software, Visualization, Writing – review & editing. XW: Data curation, Writing – review & editing. YH: Visualization, Writing – review & editing. HW: Data curation, Writing – review & editing. SL: Methodology, Writing – review & editing. AS: Writing – review & editing. SZ: Data curation, Investigation, Writing – review & editing. AK: Writing – review & editing. PH: Writing – review & editing. MA: Funding acquisition, Writing – review & editing. ME: Funding acquisition, Writing – review & editing. ZZ: Funding acquisition, Resources, Supervision, Writing – review & editing. PZ: Project administration, Supervision, Writing – review & editing.
