# Supplementary material for: Insights into the ameliorative effect of ZnONPs on arsenic toxicity in soybean mediated by hormonal regulation, transporter modulation, and stress responsive genes
Source: Front Plant Sci. 2024 Jul 30;15:1427367. doi: 10.3389/fpls.2024.1427367 (PMC11319271; doi:10.3389/fpls.2024.1427367)
Supplement: Supplementary file 1 [file DataSheet_1.pdf]

Supplementary Material

Insights into the Ameliorative Effect of ZnONPs on Arsenic Toxicity in Soybean Mediated by Hormonal Regulation, Transporter Modulation, and Stress Responsive Genes

Muhammad Zeeshan[1,2] †, Chenyu Sun[3]†, Xin Wang[1], Yuxin Hu[4], Hao Wu[1], Shengnan Li[1], Abdul Salam[1], Shiqi Zhu[1], Aamir Hamid Khan[5], Paul Holford[6], Mohammad Ajmal Ali[7], Mohamed Soliman Elshikh[7], Zhixiang Zhang[1*], Peiwen Zhang[1,2*]

[1] National Key Laboratory of Green Pesticide, South China Agricultural University, 510642 Guangzhou, China

[2] Yingdong College of Biology and Agriculture, Shaoguan University, Shaoguan 512005, China

[3] College of Natural Resources and Environment, Northwest A&F University, Yangling, 712100, China

[4] College of Pastoral Agriculture Science and Technology, Lanzhou University, Lanzhou, China

[5] Department of Biogeography, Paleoecology and Nature conservation, Faculty of Biology and Environmental Protection, University of Lodz, Lodz, Poland

[6] School of Science, Western Sydney University, Locked Bag 1797, Penrith, NSW 2751, Australia

[7] Department of Botany and Microbiology, College of Science, King Saud University, Riyadh 11451, Saudi Arabia

[1] These authors contribute equally

*Corresponding authors; zdsys@scau.edu.cn (Z. Zhang); zpw1993gz@163.com (P. Zhang)

Summary table of reads from RNA-seq in the 8 libraries of AsV-stressed soybean roots in response to ZnONPs.

a

Sample	Total raw reads	Total Clean reads	Total_mapped	Unique_mapped /%	Q20%	Q30%
CK_1	58515990	57952486	53764148(92.77%)	52202985(90.08)	97.92	93.76
CK_2	52796044	51121426	47345705(92.61%)	46035836(90.05)	97.58	93.12
AsV_1	50832550	49648720	44006087(88.63%)	42715597(86.04)	97.92	93.91
AsV_2	54146966	53017064	47509322(89.61%)	45968655(86.71)	97.62	93.21
ZnOA1	54463158	53440464	49508597(92.64%)	48024913(89.87)	97.93	93.99
ZnOA2	57685508	55865610	51548861(92.27%)	50073400(89.63)	97.84	93.82
ZnOB1	54290098	53596846	49349020(92.07%)	47837913(89.26)	97.95	93.97
ZnOB2	59833156	58964568	54331347(92.14%)	52526903(89.08)	97.97	94

b

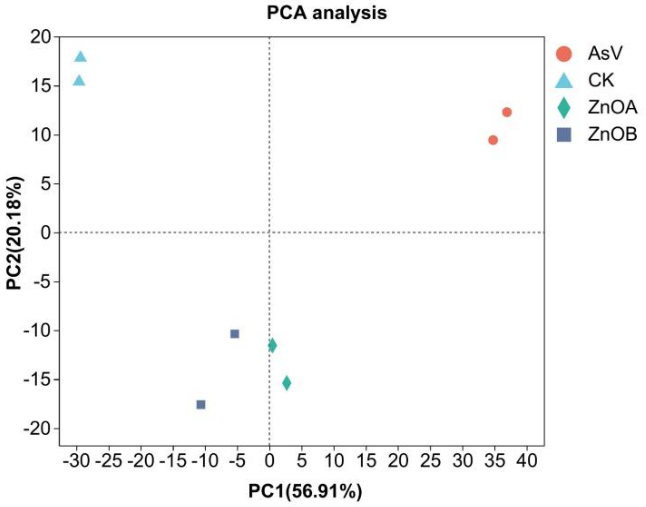

Figure S1. Summary of the sequence data and relationship of different treatments. (a) Summary table of reads from RNA-seq in the 8 libraries, (b) Principal component analysis of 8 libraries showing variation among the treatment's groups.

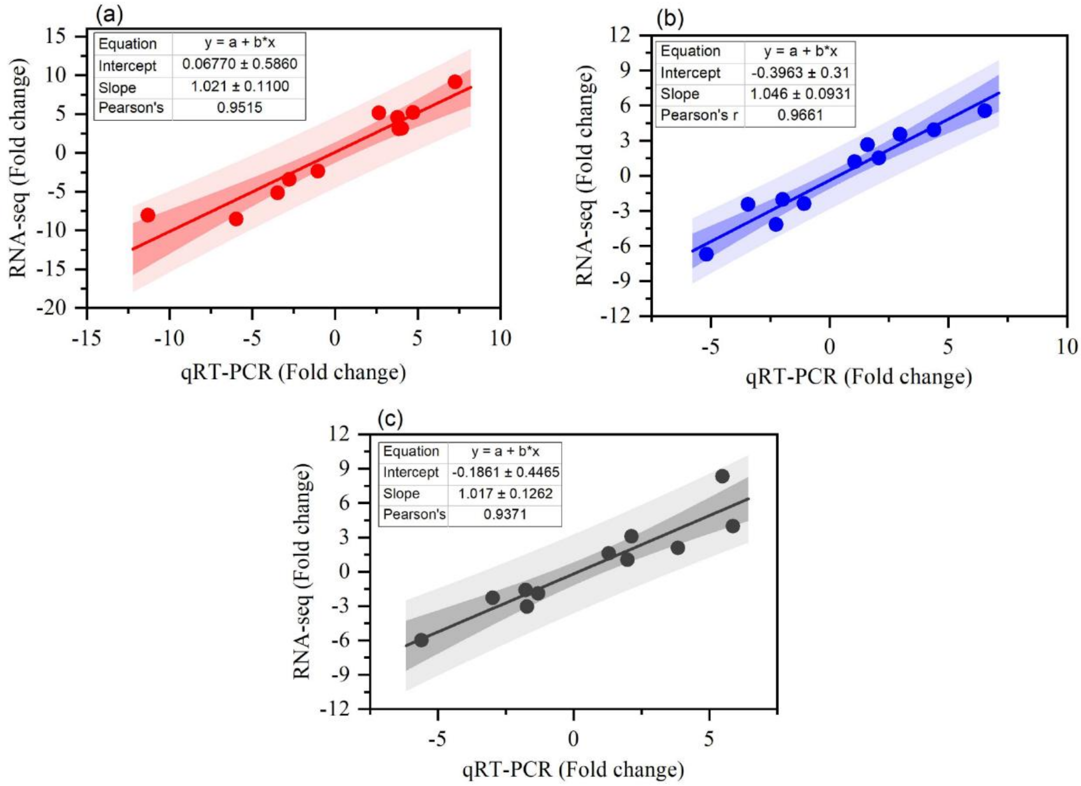

Figure S2. Regression correlation of expression level changes of eleven randomly selected DEGs (a) Ck_vs_AsV, (b) Ck_vs_ZnOA and (c) Ck_vs_ZnOB analyzed by qRT-PCR (x-axis) and RNA-seq (y-axis).

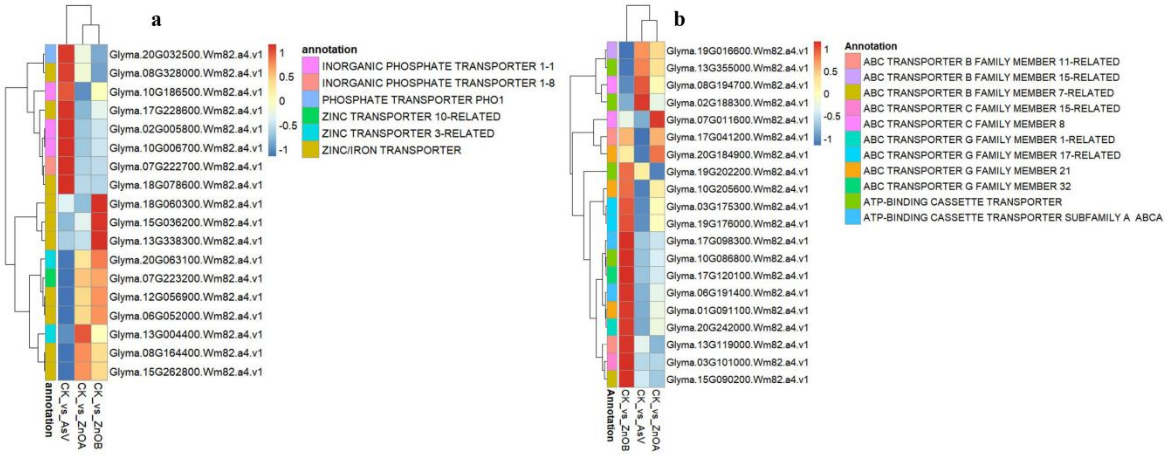

Figure S3. ZnONPs-modulated transporters DEGs under As stress in soybean roots. a and b represent the heatmap of DEGs encoding *inorganic phosphate transporter* (*PHT*), *zinc/iron transporter* (*ZIP*) and *ATP binding cassete* (ABC) transporter. The scale represents normalized log2 fold change values.
